# High hopes? Precision psychedelic addiction medicine

**DOI:** 10.3389/fpsyt.2025.1681795

**Published:** 2026-01-28

**Authors:** Rayyan Raja Zafar, Patrick Kleine, Danielle Kurtin, Matthew Wall, David Erritzoe

**Affiliations:** 1 Centre for Psychedelic Research and Neuropsychopharmacology, Division of Psychiatry, Department of Brain Sciences, Imperial College London, London, United Kingdom; 2 Perceptive, London, United Kingdom

**Keywords:** addiction, EEG, fMRI, PET, psychedelic, biomarker, precision psychiatry

## Abstract

Despite decades of neuroscience research and significant investment in addiction neuroimaging, clinical outcomes for individuals with substance use and behavioural addictions remain poor. Only 1.8% of people with substance use disorders receive effective treatment, highlighting a major disconnect between mechanistic understanding and clinical utility. This paper calls for a reorientation of addiction neuroscience, from a predominantly diagnostic focus toward a theragnostic framework, in which biomarkers are used to stratify patients, guide treatment decisions, and predict outcomes. We argue that the integration of translational neuroimaging biomarkers, particularly fMRI, EEG, and PET, within psychedelic addiction research offers a unique and timely opportunity to catalyse this shift. Psychedelic compounds such as psilocybin represent a new class of therapeutics capable of engaging neuroplasticity, reward and emotional processing, and cognitive control networks central to addiction pathophysiology. We review how acute and pre–post neuroimaging paradigms can index pharmacodynamic effects and longer-term treatment response and propose a roadmap for embedding biomarkers in early and late phase clinical trials. Drawing on ongoing studies at the Centre for Psychedelic Research at Imperial College London, we outline how multimodal biomarkers are being co-developed alongside clinical trials in gambling and opioid use disorders to identify biotype-specific responses and build a deeply phenotyped treatment population. We argue that these biomarkers, if validated, could serve as regulatory-grade tools for drug theragnostic co-development, mirroring successful models in oncology and neurology. Importantly, we emphasise that realising this vision will require robust multi-stakeholder collaboration, including academia, industry, regulatory agencies, funders, healthcare systems, and patient groups alongside dedicated investment to build a scalable theragnostic infrastructure for addiction research and medicine. In conclusion, psychedelic therapy offers more than symptomatic relief, it presents a vehicle for transforming how we diagnose, treat, and understand addiction. By embracing theragnostic principles and prioritising biomarker integration, addiction medicine has the potential to move towards personalised and precision-guided care.

## 1 Biomarker inertia in addiction – the story so far

Despite decades of neuroscientific investigation and billions invested in brain imaging, clinical outcomes in addiction remain staggeringly poor. A recent WHO report estimates that only 1.8% of individuals with substance use disorders (SUDs) globally receive effective treatment (1). This figure is particularly sobering when set against the backdrop of nearly 40 years of neuroimaging research in addiction, much of which has yet to yield actionable tools for clinical use (2).

To date, the field has produced 479 imaging paradigms across 409 distinct protocols, the vast majority relying on human functional magnetic resonance imaging (fMRI) (2). Notably, over 70% of human addiction brain biomarkers studies have been aimed at diagnostic classification, with task-based cue-reactivity emerging as the lead candidate for a regulatory-approved biomarker of addiction (3). Yet the diagnostic yield of this work has been minimal. Few, if any, biomarkers have been translated into routine clinical practice or drug development (2). Efforts to improve translation from preclinical to clinical biomarkers are however underway and opportunities to improve this translational bridge are emerging and are discussed more in depth here but are beyond the scope of this review (4).

This disproportionate focus on diagnostic biomarkers has created a stagnation in the field of addiction neuroscience. What is urgently needed now is a redirection toward developing translational and theragnostic biomarkers (for definitions, see **Table 1**) to advance therapeutic development and a move away from basic science experiments that do not directly serve to advance treatments. Shifting the aim of addiction neuroimaging biomarker research from predominantly diagnostic classification to theragnostic development can provide clinical value in four key ways: (1) catalyse the discovery of novel therapeutics; (2) inform biotype-specific treatment assignment; (3) move beyond DSM-defined diagnoses towards biologically meaningful patient stratification and biotyping; and (4) provide predictive or prognostic indicators to optimise treatment outcomes. This shift would align addiction medicine with a broader trend across psychiatry and indeed across the whole of medicine towards precision-guided, mechanism-based approaches (5, 6).

**Table 1:** Categories of biomarkers relevant to clinical drug development pipelines.

Biomarker type	Definition
Diagnostic	A biomarker that can differentiate individuals with addiction disorders from those who engage in substance use or behaviours (e.g., gambling, internet use) but have not developed an addiction disorder or can distinguish clinically relevant subtypes of addiction disorders.
Early/Late Phase Clinical Trial Response	There are early and late phase clinical trial functional response markers that have been utilised in addiction drug development (reviewed below). These markers can be integrated into a FAST-FAIL approach to help with go/no-go decisions on advancing therapeutics through stages of development.
Acute, Sub-acute and Long-term Response	There are also acute, sub-acute and longer-term functional response markers which are responsive to treatment, and these can provide core evidence for the mechanism of action of the therapeutic. Data generated from these markers is useful for developing precision and personalised approaches to treatment.
Prognostic	This is a biomarker that can indicate the future trajectory of an individual’s disorder towards relapse or remission.
Predictive	A biomarker that can predict the clinical response of an individual to an intervention.
Theragnostic	A combination approach utilising diagnostic or prognostic biomarkers to guide patient intervention selection.
Target Engagement	Target engagement, in the context of drug discovery and pharmacology, refers to the direct interaction or binding of a drug or other chemical compound with its intended protein target within a living system. This can be conceptualised as a biomarker of likely drug efficacy.

Encouragingly, early work in this space suggests that neuroimaging biomarkers may, in some cases, outperform clinician judgement in predicting treatment response, and some studies have begun to assess how neuroimaging data can influence or moderate treatment outcome, with some replication studies beginning to validate this approach (7, 8). However, a major recent systematic review on neuroimaging biomarkers in addiction suggests less than 5% of studies adopt the strategy of trying to understand the predictive or prognostic potential of these markers, and no validated theragnostic tools currently exist for addiction therapeutic development (2).

This perspective calls for a reorientation of addiction neuroscience research over the next decade. We feel it is timely to refocus efforts from diagnostic classification to a translational, precision-medicine framework guided by biomarker discovery and validation. Particularly, this perspective is the first to our knowledge to integrate multimodal imaging into a theragnostic framework for biomarker-guided psychedelic addiction medicine.

To anchor these arguments in practical application, we highlight ongoing psychedelic therapy (PT) research at Imperial College London, specifically focused on psilocybin as an investigational therapeutic for addiction. We show how this work integrates neuroimaging biomarkers within experimental and translational medicine frameworks to accelerate both drug development and mechanistic understanding that could build the foundations for precision psychedelic medicine. It is our proposal that this convergence of neuroscience, biomarkers, and these novel and highly promising therapeutics holds the greatest potential for transformative change in addiction treatment and validated biomarker development over the next decade.

It is the hope that this perspective combines mechanistic insights into both psychedelic research and biomarker discovery in addiction, bridging these as yet disparate domains and providing a translational roadmap for mechanism-based, biomarker-informed psychedelic research.

## 2 Drug development

The process of drug development traditionally begins with the identification of a biological target implicated in a given disease process. This is followed by high-throughput screening of candidate molecules, evaluating their binding affinity, bioavailability, and pharmacological profiles *in vitro*. A refined subset is then progressed into preclinical animal models, where safety, toxicity, pharmacokinetics (PK), pharmacodynamics (PD), and target engagement are assessed *in vivo*. Lead compounds that meet these criteria advance to early-stage human trials (9).

When considering the preclinical addiction drug development approaches, a variety of *in vivo* animal models exist to assay mechanisms and the potential of candidate compounds (10). However, capturing the nuanced behavioural and environmental complexities of addiction in animal models has proved difficult, and translating observations from preclinical animal models to humans has had limited, albeit some, success (11, 12).

Conversely, preclinical psychedelic drug development has benefitted from back-translation of human neuroimaging to determine occupancy of psychedelics in rodent models, which has benefitted a more optimised and targeted development of compounds, including novel second and third generation psychedelics and non-psychedelic analogues (13). Indeed, harmonising approaches across preclinical and clinical neuropsychopharmacology will provide a deeper understanding of the molecular and functional mechanisms of drug action, and this approach presents a unique opportunity for addiction psychedelic drug development, of which some companies (Alvarius Pharmaceuticals) are now spearheading. A recent review highlights how the adoption of such translational assays is critical for the interpretation of animal studies of psychedelic effects on cognition, affect and reward and how these studies have the potential to help unravel the mechanisms which contribute to their therapeutic effects, but only if they involve relevant doses (14).

In parallel to aligning preclinical and clinical mechanistic research on interventions, ongoing human multimodal research into the development of validated, reliable and scalable biomarkers in addiction remains essential. Importantly, though this review focuses primarily on neuroimaging biomarkers, it is imperative to test and consider the association between CNS and non-CNS markers relevant to addiction too, as research demonstrates the important role of the involvement of peripheral markers. Despite recent advances in identifying candidate biomarkers of addiction across neural, plasma-based, genetic and epigenetic domains, no single measure has yet demonstrated sufficient clinical or translational utility when used in isolation. While some show promise in substance use and behavioural addictions, the complexity and heterogeneity of addictive disorders demand a more integrated approach. Multimodal research that combines multiple biological systems, psychological testing and behavioural markers offers a more realistic path forward, not only for improving biomarker precision but also for individualised risk profiling, for guiding treatment selection and monitoring therapeutic response (15). The future of addiction medicine lies in embracing this complexity, rather than oversimplifying it.

To date, neuroimaging biomarkers have been the most studied biomarker in addiction drug development. Studies that clarify developmental trajectories, neurocircuit dysfunction, and molecular targets in addiction are crucial to the rational design of new therapeutics (16). This, along with advances in large-scale genetic consortia (e.g., the Psychiatric Genomics Consortium), digital phenotyping, and biomarker technologies, are converging to define a new era of precision psychiatry (17) of which addiction medicine could greatly benefit. These tools are not only shaping therapeutic design but are increasingly integrated into clinical trials to support stratification, enrich for likely responders, and guide outcome prediction as reviewed here (18).

Increasingly, insights from genetics, digital phenotyping, and multimodal biomarkers are converging to define a new era of precision psychiatry. These tools are not only shaping the design of more targeted compounds but are also supporting clinical trial enrichment and mechanism-based therapeutic discovery with multistakeholder precision psychiatry roadmaps now being adopted across the EU and North America for mental health conditions more broadly (19, 20).

**Figure 1:**
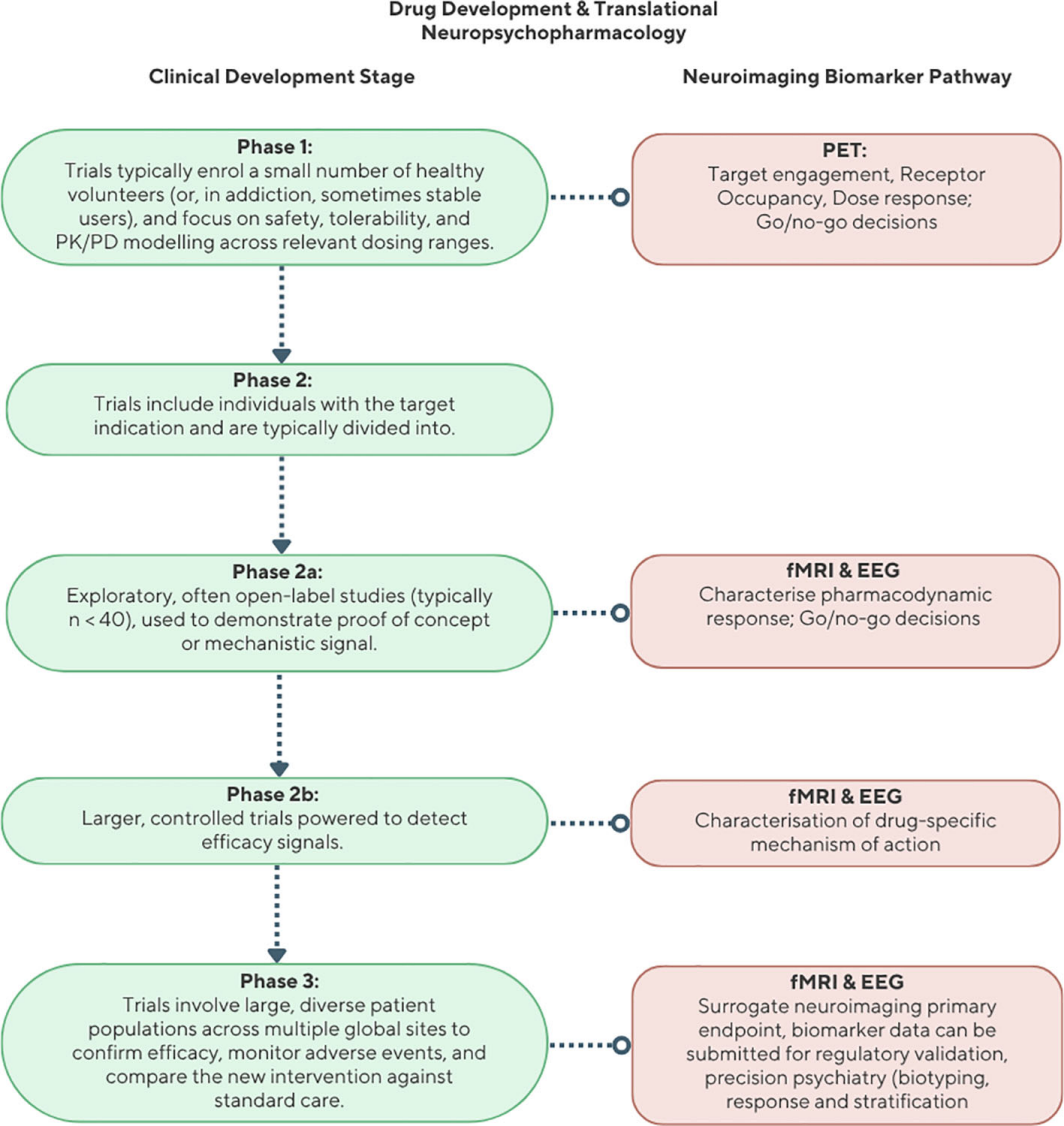
Structured phases of the clinical development pipeline.

At each clinical trial phase (**Figure 1**), neuroimaging-informed, brain-based biomarkers [e.g., electroencephalography (EEG), functional magnetic resonance imaging (fMRI), and positron emission tomography (PET)] can play a critical role in assessing pharmacodynamic responses, establishing target engagement, and discriminating treatment responders from non-responders (21). When integrated into early-phase study design, these markers can also inform ‘go/no-go’ decisions and reduce the high failure rates that characterise psychiatric drug development. This is particularly relevant in addiction and psychiatry, where nearly three-quarters of investigational compounds fail to progress beyond Phase 2 (22, 23). Poor mechanistic understanding, clinical heterogeneity, and inadequate biomarkers all contribute to these failures. Efforts to incorporate experimental medicine frameworks with embedded mechanistic endpoints are gaining traction across psychiatric trials, including in initiatives such as the NIH’s Experimental Therapeutics Approach and the European Medicines Agency’s Biomarker Qualification Programme (24, 25).

## 3 Neuroimaging in drug development

Translational neuropsychopharmacology leverages human neuroimaging to bridge the gap between basic neuroscience and clinical treatment development. In addiction research, neuroimaging has been pivotal in advancing our understanding of the pathophysiology underlying key features of addiction (such as disrupted reward, salience, executive, and affective processing), and identifying potential biomarkers that may serve as targets or readouts for novel therapeutics (2, 26).

Integrating imaging tools into early- and mid-phase clinical trials facilitates the evaluation of the mechanistic effects of new interventions, including psychedelic compounds. Linking brain-based biomarkers with clinical and behavioural outcomes offers a richer and more precise account of how interventions act on the neural substrates of addiction. Such approaches have been successful in identifying putative predictive and prognostic psychedelic biomarkers in depression (27, 28). There are many ways to implement this approach in addiction medicine, such as integrating functional, structural, and molecular neuroimaging biomarkers with peripheral metrics, psychometric, behavioural and neurocognitive assessments (29).

Importantly, neuroimaging biomarkers can also support regulatory decisions by offering mechanistically grounded evidence of treatment effects, as was seen in the case of the FDA approval of Alzheimer’s drug aducanumab (30). As the field begins to shift towards mechanism-of-action-driven trial design, particularly in early-phase drug development, neuroimaging provides a means to test whether a candidate agent is engaging its intended target, altering disease-relevant circuitry, and producing measurable change in brain function.

These approaches are not hypothetical. Numerous working groups, including those supported by the National Institute on Drug Abuse (NIDA), the European College of Neuropsychopharmacology (ECNP), and international consortia such as ENIGMA-Addiction, have called for the systematic development of neuroimaging biomarkers to guide precision medicine in addiction and psychiatry at large. A white paper co-authored by 30 global leaders in addiction science laid out a translational roadmap to incorporate neuroimaging in future treatment trials, specifically advocating for their use in drug development and regulatory qualification processes (31, 32).

Despite these advances, industry uptake has lagged. To date, few neuroimaging biomarkers have been formally qualified by regulators for psychiatric indications, and even fewer are embedded in addiction drug development programmes. Strengthening academic-industry partnerships, improving standardisation across imaging pipelines, and demonstrating clinical utility through prospective trials are critical next steps.

In this context, psychedelic research offers a promising testing ground for embedding neuroimaging into translational pipelines (27). These compounds act rapidly, target specific receptor systems (e.g., 5-HT2A), and induce measurable changes in brain function and subjective experience, making them ideal for the development of pharmacodynamic biomarkers (33). In the following sections, we examine how fMRI and EEG have been used to support drug development in addiction and how these modalities can be deployed in experimental medicine studies to guide the advancement of psychedelic therapies.

### 3.1 FMRI & EEG in translational addiction research

FRMI and EEG have been central to efforts in identifying functional brain changes associated with addiction and, increasingly, in evaluating the effects of pharmacological and behavioural interventions. These modalities offer distinct yet complementary insights. FMRI captures haemodynamic changes via the blood oxygenation level dependent (BOLD) signal linked to neural activity with relatively high spatial resolution and the ability to image deep brain structures. EEG provides high temporal resolution data of electrophysiological brain dynamics (6, 16, 31).

In this section, we summarise how fMRI and EEG are currently being employed in early- and later-phase clinical trials. In early-phase clinical trials, biomarkers can inform whether target engagement corresponds with changes in relevant physiological processes. Late-stage clinical trials may also benefit from using fMRI and EEG biomarkers to inform secondary or exploratory endpoints, as well as provide evidence of treatment-induced brain changes. Given the considerable resources required to run mid- to late-stage clinical trials, candidate compounds that modulate neuroimaging biomarkers in phase 1b/2a provide promising signals for investment from drug developers.

#### 3.1.1 Neuroimaging in early- and late-phase clinical trials in addiction

##### 3.1.1.1 fMRI

In early-phase trials, fMRI can be used as a treatment response biomarker. Its purpose lies in the identification of functional central nervous system (CNS) effects resulting from pharmacological interventions pertinent to the compound’s mechanism of action and/or the intended target population (34). Although not inherently indicative of direct target engagement, as can be determined with PET imaging, an fMRI signal can indirectly imply such engagement if a biologically plausible correlation can be established between the observed fMRI response and the molecular target being pursued due to the spatial correspondence between functional MRI activation/connectivity maps and the regional distribution of molecular targets (e.g., receptor density or gene expression) (35). The NIMH FAST-FAIL experimental therapeutics initiative offers a framework for utilising fMRI to identify the success (or lack thereof) of target engagement. In the FAST-FAIL approach, early-phase compounds are required to demonstrate a pre-registered biomarker change as a condition for further progression in the development pipeline (36). Within this framework, early-phase experimental agents are mandated to induce predetermined biomarker modifications (including fMRI markers), as outlined in preregistered trial protocols. Failure of the compounds to elicit the specified alterations results in the exclusion of these agents from advancing to subsequent trial phases (37). This approach has been used previously in a trial of alcohol use disorder (AUD) where positive fMRI results demonstrated the positive effects of one agent (varenicline) on reducing cue-reactivity, which influenced the decision to pursue further development, subsequently demonstrating clinical efficacy in mitigating alcohol cravings (38, 39). Conversely, null early-phase fMRI findings for a different AUD agent (pexacerfont) prompted a redirection of attention toward a competitor agent (verucerfont) (40).

Our group at Imperial College London has pioneered the use of acute fMRI to evaluate pharmacological challenges with early-stage addiction treatments, aiming to characterise the neuropharmacology of relapse pathways in cocaine, alcohol, opiate-dependent, and healthy individuals in the ICCAM study (41). Using a placebo-controlled, randomised, crossover design, our team assessed modulation of reward, impulsivity, and emotional reactivity across tasks probing incentive processing, inhibitory control, and affective response, employing selective antagonists targeting µ-opioid (naltrexone), dopamine D3 (GSK598809), and neurokinin 1 (vofopitant/aprepitant) receptors, establishing pharmacological fMRI as a useful platform to assess the mechanism of action and efficacy of early stage compounds (41, 42).

FMRI-informed biomarkers are increasingly utilised in late-stage clinical trials. A 2021 systematic review identified over 100 Phase 3 or 4 clinical trials that incorporated fMRI as part of their design, representing approximately 21% of all drug intervention trials with neuroimaging components. This growing trend highlights the increasing value placed on brain-based endpoints in advanced-stage drug development (43). Despite this, at present, there is only one fMRI-identified biomarker under consideration with the European Medicines Agency; a battery of brain MRI measurements that includes two task fMRI paradigms and a resting state acquisition, with a goal of enriching trials in autism spectrum disorder (44).

Applying a similar framework to addiction research could yield substantial benefits. fMRI cue-reactivity, one of the most well-studied tasks in addiction neuroimaging, has been proposed as a viable biomarker for predicting relapse and treatment outcomes. A recent systematic review outlined a potential pathway for regulatory validation of this task under current biomarker development frameworks (3). With sufficient replication and standardisation, fMRI cue-reactivity measures could be used to enrich trials by identifying patients with heightened neural responses to drug cues, i.e. those potentially more likely to benefit from specific interventions.

Despite the promise of fMRI cue-reactivity measures, uptake within the pharmaceutical industry has been limited. Bridging this gap will require stronger partnerships between academia and industry, collaborative validation efforts, large-scale data sets and real-world demonstration of clinical utility. Importantly, this also entails developing scalable, robust, and cost-effective imaging hardware and analysis pipelines that can be deployed across diverse sites and populations.

##### 3.1.1.2 EEG

In a comparable manner to fMRI, metrics derived from EEG [such as event-related potential (ERP)] index key addiction-implicated processes and show potential for identifying biomarkers to addiction drug development. The strong test-retest reliability of EEG-derived metrics makes them reliable assessment tools for repeated monitoring of the effects of candidate molecules on neurophysiological measures of addiction-relevant cognitive domains and brain dynamics (45–48). Studies have demonstrated addiction-relevant ERP measures with potential sensitivity to pharmacological and behavioural interventions across addiction disorders (49). Examples of ERP measures of addiction-relevant domains include the following: salience attribution (cue reactivity P3/Late positive potential), cognitive/attentional processing (oddball P3), and response inhibition (No-Go N2). See the reviews of Bel-Bahar et al. (49), Houston and Schlienz (50), and Campanella et al. (45) for detailed summaries.

Equally, resting state EEG power spectra, beta, and possibly delta, theta, and alpha power show sensitivity to treatment/abstinence in addiction populations, and therefore could be employed in a similar fashion to measure functional target engagement (49, 51, 52). Although additional validation is required, utilising such markers may provide indications about a compound’s neurobiological effects on core addiction-related processes at an early stage of development and used as a decision point. Such an approach has been successfully done in early-stage depression trials using EEG (22), where EEG measures derisk candidate compounds for progression into later-stage clinical trials (53). Moreover, the utilisation of EEG in later-stage clinical trials benefits from low cost, ease of use, and wide availability.

Validated EEG/ERP markers that indicate sensitivity to interventions in early phase trials should be carried through into later stages where possible (22). There is evidence that baseline ERP cognitive oddball P3 (54–56), Go/No-Go No-Go P3 difference (54, 57), and Cue-elicited LPP (58); and resting state EEG theta power (59, 60) can predict treatment/abstinence outcomes in addiction disorders. There is potential for these measures to enrich later phase clinical trials through patient stratification, informing selection of sub-populations that are more likely to be responders to the candidate intervention, improving clinical translation and pushing forward potential personalised treatment paradigms when combined with machine learning approaches and integrating across multimodal markers previously described (61).

With EEG likely to be a more accessible technology in the clinic for now, such markers, if robust and scalable, may have utility outside of academia and translate to the clinic with administration to patients in real-world clinical settings. Indeed, there are signs that there is some, if limited, uptake of these methods by addiction medicine clinicians in the United States. These tools theoretically could eventually be used to define drug on-label populations (22).

##### 3.1.1.3 Simultaneous/combined PET/fMRI

Integration of data from PET target occupancy studies and fMRI and EEG methods provides the most comprehensive strategy for relating information on drug–target interactions directly with a measure of functional effects in the brain. Some studies have adopted this approach, with one assessing the extent of binding of an antagonist to its μ-opioid target with modulation of fMRI reward responses to the administration of a palatable food stimulus in healthy volunteers (62). This study validated the target as a modulator of satiety responses in humans, and the PET data indicated an appropriate pharmacological dose range based on both the measures of target interaction and the pharmacodynamic effect, demonstrating the clear translational utility and benefit of this approach in drug development. The advent and wider availability of novel simultaneous PET-fMRI technologies will greatly advance such research and could represent a useful technological advance for therapeutic development in addiction (63).

An important yet underutilised avenue in both addiction drug development and the advancement of precision psychedelic medicine is the use of PET imaging to measure dynamic neurotransmitter release. Although such approaches can be costly and involve dosimetry and scan burden challenges, they uniquely enable *in vivo* assessment of synaptic function and, particularly, the restoration of perturbed neurotransmission, which is seen in addiction, following therapeutic intervention, these methods have been reviewed in depth here (29, 64). This is especially relevant in addiction, where dysregulation of dopamine, GABA, opioid, and serotonin (5-HT) systems has been demonstrated (65–67). Transmitter release paradigms using PET (e.g., amphetamine challenge or pharmacological displacer studies) have been instrumental in mapping these disruptions and evaluating candidate therapeutics (68). Incorporating such measures into early-phase addiction psychedelic trials, especially as mechanistic or exploratory biomarkers, could provide critical insight into the direct molecular-to-functional mechanisms of the drug. Extensive work from our group and others offers a valuable framework for how these methods might be deployed to bridge neurobiology, clinical response, and drug development pipelines.

Together, these developments outline a translational roadmap for neuroimaging research to identify biomarkers optimising drug development across clinical trial phases. How can precision psychedelic addiction medicine follow this path?

## 4 Translational biomarkers in psychedelic drug development

Neuroimaging has played a foundational role in contemporary psychedelic research. A wide range of acute and longitudinal studies have investigated how serotonergic psychedelics alter brain dynamics, modulate neural circuits, and drive therapeutic outcomes. These approaches have helped define the *psychedelic brain state*, which is characterised by distinct patterns of functional reorganisation, desynchronisation, increased signal complexity, and altered connectivity (33).

To date, such measures have been sparsely used in psychedelic drug development outside of academia. However, there has been a strong case made for the utility of imaging to help guide drug development and precision and personalised psychedelic medicine (27, 69).

**Figure 2:**
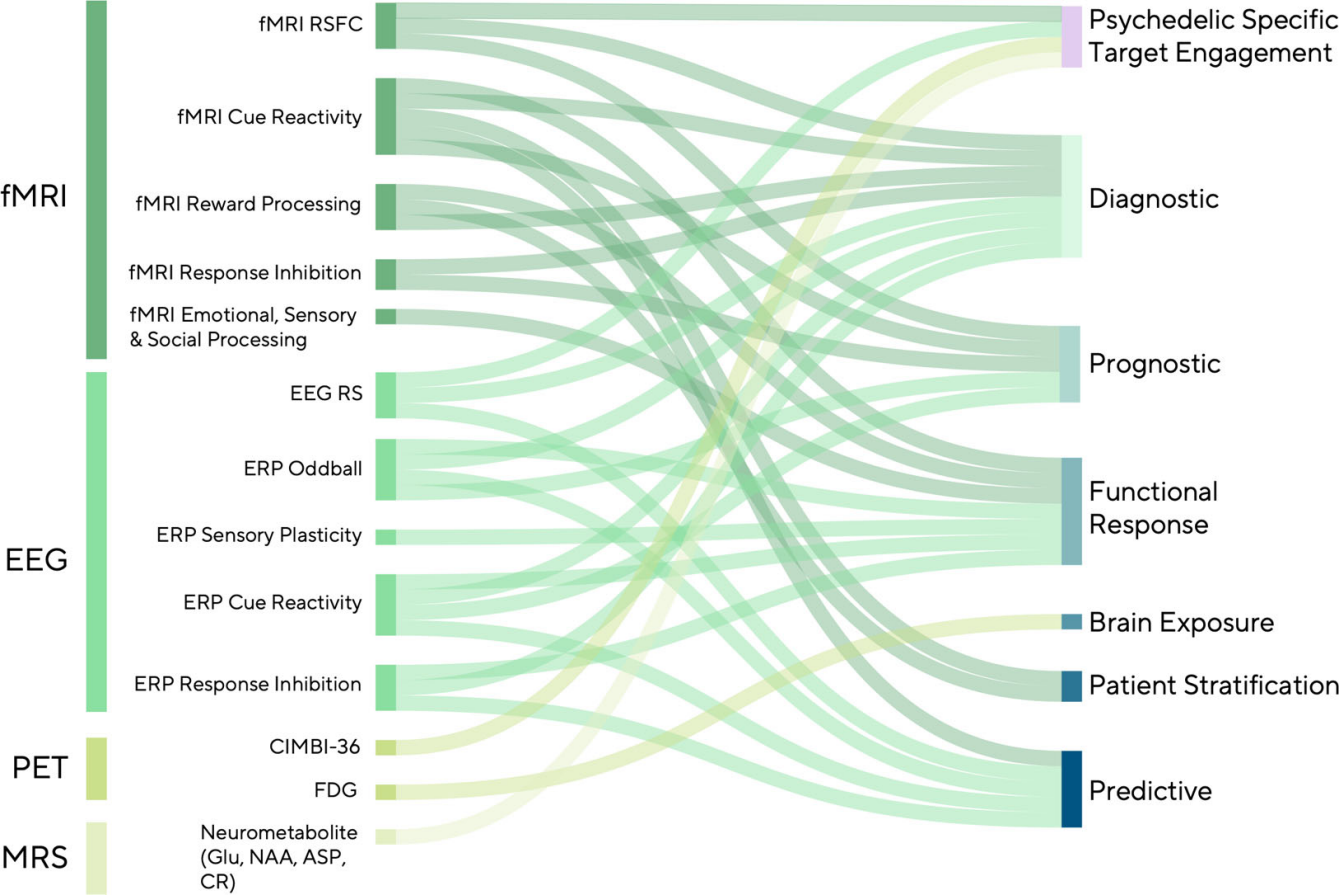
Candidate psychedelic addiction medicine neuroimaging biomarkers.

In this section, we highlight emerging biomarkers from psychedelic EEG, fMRI, PET, and magnetic resonance spectroscopy (MRS) studies and discuss how they can inform drug development pipelines by indexing target engagement, brain exposure, and treatment response more broadly. We also illustrate how these tools may enable stratification and biotype-specific treatment selection, which are key priorities for theragnostic implementation (**Figure 2**).

### 4.1 Acute imaging biomarkers in psychedelic research

The 5-HT2a receptor (5HT2AR) is the primary target of serotonergic psychedelic drugs (e.g., LSD, DMT, and psilocybin). Concurrent administration of psychedelic drugs during EEG (**Box 1**) and fMRI (**Box 2**) has revealed markers of functional pharmacodynamic target engagement.

Box 1 **Putative acute resting state EEG psychedelic 5-HT2AR target engagement biomarkers**

1. Acute resting state eyes-closed alpha power suppression following administration of DMT (70–72), psilocybin (73–75) and LSD (76) is the most robustly observed EEG marker of psychedelic agonism at the 5-HT2A receptor.
2. Elevations in Lempel Ziv Complexity (LZ), a measure of signal diversity, following LSD, DMT, and Psilocybin administration have been repeatedly observed across pharmaco-EEG psychedelic studies (70–72, 75, 77, 78). 
3. Alpha Power and LZ EEG measures both associate with real-time measures of subjective intensity and blood plasma (71, 72), therefore are promising biomarkers of real-time target engagement, with significant applicability in early stages of psychedelic compound development.
4. Preliminary findings indicate acute EEG LZ at 1– and 2-hours post dose predict long-term response to psilocybin. Specifically, improved psychological well-being at 1 month following a single 25mg dose of psilocybin in naïve healthy participants (77). However, thus far, this analysis is yet to be conducted in clinical populations.
5. Event-related oscillatory (ERO) activity approaches have been found to be useful in explaining psychedelic mechanism of action in pre-clinical models of addiction, and similar approaches could be translated into humans, to better integrate pre-clinical and human biomarker approaches, e.g., (79).

Box 2 **Putative acute resting state fMRI psychedelic 5-HT2AR target engagement biomarkers**

1. Decreased within-network connectivity, especially in the default mode and salience networks (71, 80–83).
2. Increased between-network and global connectivity, suggesting network desegregation and reorganisation (71, 76, 81–86).

*NB: A myriad of preprocessing and analytical approaches have been applied across psychedelic rs-fMRI publications to date that can impact the directionality of these measures, highlighting the need for a standardisation of approach within the field* (87).

Recent methodological advances, such as Receptor-Enriched Analysis of functional Connectivity by Targets (REACT), allow researchers to map functional connectivity changes to underlying receptor distributions, including the 5-HT2A receptor. This represents a powerful strategy for linking pharmacological action with circuit-level changes, with this technique proving useful in decoding the receptors responsible for the acute pharmacological effects of psychedelic drugs on brain networks (88).

Moreover, psychedelic-induced changes in neurometabolite concentrations (glutamate, GABA, and others) can be measured with Magnetic Resonance Spectroscopy (MRS) (89–91) and psychedelic 5-HT2AR occupancy-dose, and occupancy-subjective effect relationships can be quantified with CIMBI-36 PET ligand studies (92–94). Together, these EEG, fMRI, MRS, and PET prospective psychedelic-specific target engagement biomarkers could be employed in psychedelic drug discovery to deeply phenotype a candidate compound’s pharmacodynamic properties to infer engagement at the target(s) of interest. These approaches will prove particularly useful for second and third-generation psychedelics as well as other non-psychoactive 5-HT2A agonist compounds in development (95).

Moving beyond brain networks to specific brain functions, there has also been an increased interest in modelling the effects of the psychedelic state on several neural processing domains. Previous work has employed cognitive (96–98); emotional processing (99–105); social processing (90, 106); self-other processing (106, 107), sensory processing (96, 97, 108–110); visual processing (111); response inhibition (112) and autobiographical memory (113) paradigms acutely to understand psychedelics impact on functionally relevant neurobiological and cognitive domains, demonstrating sensitivity of these tasks to psychedelics.

Such paradigms could be used in acute drug challenge studies as functional response biomarkers to understand how a candidate molecule may acutely target addiction-relevant behavioural and clinical circuits; these approaches being similar to those used in the ICCAM study (41). However, it is important to note that some tasks may not be suitable or appropriate for psychedelic studies due to their inherent psychoactive and consciousness/perceptual-altering properties, as described under acute administration of psilocybin with fMRI (86). Nonetheless, there may be useful signals from these acute imaging biomarkers which can be useful in drug development in the decision to take forward candidate compounds from healthy humans into clinical populations and to act as potential predictive markers.

#### 4.1.1 Pre–post biomarkers in psychedelic research

While acute imaging biomarkers are essential for indexing immediate pharmacodynamic effects, pre–post neuroimaging paradigms offer insights into the subacute and longer-term neural changes associated with therapeutic outcomes. These approaches may yield prognostic and predictive biomarkers that reflect enduring shifts in functional connectivity, emotional processing, and neural plasticity, which are thought to be hallmarks of the therapeutic response to psychedelics (**Box 3**).

Box 3 **Pre–post psychedelic measures of sub-acute and long-term neural changes**

1. Task-based fMRI studies have demonstrated changes in emotional processing after psychedelic therapy. For example:
  • In healthy individuals, psilocybin-induced modulation of amygdala reactivity to emotional stimuli has been observed at 1- and 4-weeks post-treatment (114).
  • In individuals with major and treatment resistant depression, amygdala responsivity to emotional faces may be a useful pre–post marker (115, 116).
  • Sensory processing tasks (music listening) can index functional brain changes 3 weeks post psilocybin (117).
2. fMRI can capture pre/post psychedelic changes in resting state functional connectivity and DTI measures (28, 89, 118) with possible potential to predict symptom outcome in depressed populations (119) though to date there has been no studies published utilising this approach for other psychiatric disorders including addiction, demonstrating an important and obvious research gap.
3. EEG paradigms indexing sensory neuroplasticity processes (120), can be used to measure longer-term changes in functional neuroplasticity following psychedelic dosing and then be useful to predict a relationship with clinical outcomes (97, 121). Notably we have used these tasks in completed studies of patients with chronic pain (122), anorexia nervosa (123), and obsessive compulsive disorder (124).

To date, most pre-/post-psychedelic imaging studies have focused on affective disorders, but the frameworks developed can be readily extended to addiction. These paradigms typically involve baseline imaging, followed by assessments at one or more timepoints post-treatment (e.g., 24 hours, 1 week, 1 month), allowing researchers to track longitudinal changes across relevant neural circuits. A summary of key biomarker findings, including pre-/post-studies with psilocybin and other psychedelics, has been extensively reviewed here (125).

Despite promising findings, studies assessing pre-/post-biomarker changes in addiction remain scarce, and this is an area requiring considerably more investigation. However, a recent psilocybin trial in AUD provided preliminary evidence of psilocybin’s effect on salience attribution with a cue-reactivity task, demonstrating how a cue-reactivity fMRI paradigm can provide insight into psychedelic-mediated functional changes in addiction-implicated regions associated with craving (126). To our knowledge, this is the first and only preliminary assessment of the impact of psychedelic therapy on human reward system dysfunction in addiction.

We recommend implementing these neuroimaging measures, such as those described above, in early and middle-stage phase 2a and 2b trials, to understand for the first time 1) mechanisms of action of psychedelic therapy, 2) treatment-related prognostic and predictive biomarkers, and 3) biotype-specific responding.

These neuroimaging measures that associate with or predict indication-relevant clinical outcomes, including drug use, gambling use, craving and core addiction-related processes, can be modelled at the neural level by tasks featuring inhibitory control, impulsivity, affective, emotional, cognitive and reward processing. These measures can be taken forward as candidate predictive biomarkers of treatment response, separating responders and non-responders to an intervention. Further, such identified markers can then be proposed for validation using standard regulatory approaches for use in clinical and drug development settings (127) that will enable the co-development of the therapeutic and the diagnostic/prognostic markers, giving rise to the concept of a psychedelic-specific theragnostic in addiction. There are now some well-established frameworks to validate these approaches, including the FDA BEST programme (https://www.fda.gov/drugs/biomarker-qualification-program/about-biomarkers-and-qualification) as well as the aforementioned EMA biomarker validation process.

#### 4.1.2 Realising theragnostic potential in psychedelic addiction treatment

Theragnostics, an approach that synergistically integrates diagnostics/prognostics with therapeutics, has revolutionised the treatment of complex diseases such as cancer and Alzheimer’s disease, enabling precision-targeted interventions with improved outcomes and reduced costs (128, 129). In oncology, the success of biomarker-guided therapies and companion diagnostics was catalysed by President Obama’s Precision Medicine Initiative in 2015, which laid the foundation for a re-insurable model of personalised care that is now standard practice (130) and was boosted by $215 million of public investment. Psychiatry, by contrast, has yet to receive such support, in part due to the regrettably low translation of effective therapeutics into phase II research as well as political and moral reasons (23).

We believe that now is high time to challenge this situation, not least due to the mounting evidence of positive data on the efficacy and safety of psychedelic therapy in addiction (29) but also due to the concomitant advancements in biomarker research as discussed, which will profitably optimise clinical psychedelic drug development in this indication.

Precision psychiatry, underpinned by theragnostic principles, holds potential to enhance the safety, efficacy, and cost-effectiveness of psychedelic treatments. Leveraging advances in functional and molecular neuroimaging, machine learning, digital therapeutics, and wearable biosensors will facilitate the identification of who is most likely to respond to these interventions, how, and why (5). Tools from molecular biology, including bioinformatics, pharmacogenomics, and proteomics, can deepen our mechanistic understanding of how psychedelics engage neural circuits and modulate neuroplasticity across heterogeneous populations.

The deployment of theragnostics in psychedelic addiction medicine would enable the field to move beyond mere clinical assessment of relapse and symptom reduction to offering a scalable and bio-psycho-socially informed strategy to stratify patients, personalise treatment, and track therapeutic outcomes longitudinally.

As we enter the next decade, it is vital we aim to position psychiatry and addiction research within the precision medicine framework. To that end, in the forthcoming sections, we illustrate how our early phase experimental and translational research of psilocybin in addiction disorder will adhere to the four ways to achieve the aim of addiction neuroimaging biomarker research from diagnostic classification to theragnostic developments.

#### 4.1.3 CPR addiction studies

At the Centre for Psychedelic Research (CPR) at Imperial College London, we are actively developing and validating addiction-relevant neuroimaging biomarkers within our ongoing and upcoming psychedelic study programme in behavioural and substance use disorders (as these studies are still at an early stage there are currently no peer-reviewed publications presented). These efforts span both experimental medicine (mechanism-focused, early-phase) and translational medicine (linking mechanistic insights to clinical endpoints), aiming to lay the groundwork for a theragnostic approach to psychedelic addiction treatment. These studies are quite distinguishable from ongoing therapeutic trials, predominantly due to the inclusion of biomarker assessment at various timepoints.

These two complementary studies are:

1. PsiloGambling, an experimental medicine study, focusing on using fMRI and EEG to examine whether psilocybin modulates dysfunctional reward, memory, and inhibition circuits previously identified in our biomarker work. PsiloGambling is explicitly aimed at developing and validating biomarkers of target engagement and psilocybin mechanism to lay the groundwork for future therapeutic development in clinical trials (**Figure 1**, Ph2a).
2. PsilOpioid, a translational medicine clinical trial designed to assess signals of efficacy and safety in patients recently detoxified from opioid substitution therapy with opioid use disorder, incorporating secondary neuroimaging endpoints to evaluate changes in craving-related circuitry, including an fMRI cue-reactivity task, a sociality task and cognitive fMRI tasking (**Figure 1**, Ph2b).

Our focus is to begin building a biomarker framework to stratify patients, track treatment response, and inform compound development. Drawing on validated experimental tasks and biomarkers from fMRI, EEG, and peripheral sampling, we aim to characterise the neurobiological substrates of addiction and how they are modulated by psilocybin therapy. The core domains of interest include those listed in **Table 2**. Of note, recent and upcoming studies have taken a similar neuroimaging marker driven approach to clinical psychedelics addiction research (126, 134–139).

**Table 2:** Core addiction domains of interest can be indexed with various neuroimaging paradigms.

Core addiction domain	Neuroimaging paradigm
Salience attribution and cue reactivity	fMRI and EEG-based cue exposure tasks, particularly in gambling and opioid addiction. (131)
Emotion regulation and autobiographical memory	fMRI autobiographical memory, prospection, and theory of mind validated paradigms to model affective engagement and self-referential processing under psychedelics (132)
Inhibitory control and impulsivity	fMRI and EEG go/no-go and Stop Signal Tasks, critical for indexing executive dysfunction in addiction (26)
Cognitive and affective flexibility	Tasks that index adaptability in response to changing reward contingencies, central to the behavioural pathology of addiction (133)

These tasks are embedded within acute and pre-/post-therapy imaging paradigms, allowing us to examine both immediate pharmacodynamic effects and longer-term therapeutic changes.

We are also collecting peripheral blood-based biomarkers to examine genotype, metabolomics, proteomics, immune response, and collecting microbiome samples to better characterise associations between the gut and the brain. Further, we will be complementing these CNS and peripheral biological samples with wearable physiological data. The final goal of such research is to develop the most comprehensive and deeply phenotyped group of clinical patients to have received psilocybin.

This integrated, multimodal approach is designed to:

1. Identify reliable and biologically meaningful treatment response markers.
2. De-risk progression of psychedelic compounds through early-phase development.
3. Enable biotype-specific treatment selection in future trials.
4. Generate mechanistic data that can support regulatory submissions.

Our biomarker programme is structured to advance in parallel with these clinical studies, enabling the co-development of therapeutic and predictive/prognostic tools, the hallmark of a truly theragnostic model. An overview of candidate psychedelic addiction medicine biomarkers of interest leveraged in our upoming psychedelic addiction experimental and translational medicine studies is given in **Table 3**.

**Table 3:** Candidate neuroimaging biomarkers for psychedelic addiction medicine.

Candidate psychedelic addiction medicine biomarkers	
1	Cue reactivity (fMRI & EEG)
2	Acute fMRI RSFC
3	Acute resting-state EEG Alpha Power and Lempel-Ziv Complexity
4	Autobiographical Memory and Theory of Mind fMRI
5	Sociality task
6	IOWA gambling task
7	Diffusion Tensor imaging

## 5 Limitations and future directions

While the theragnostic rationale for biomarker-guided psychedelic research is compelling, several conceptual and methodological limitations currently constrain its translation. First, state-dependence presents a fundamental challenge: neural signatures identified under the acute or sub-acute psychedelic condition are not readily assessed in the same patients in follow-up states in clinical studies. This complicates interpretation of whether observed changes due to the transient pharmacological effects relate to durable therapeutic mechanisms. Longitudinal designs are required to dissociate drug-related from trait-related biomarker shifts.

Second, blinding and expectancy effects remain significant obstacles. Given the distinctive subjective experiences induced by psychedelics, full participant and investigator blinding is rarely achievable, introducing potential expectancy-related confounds (140). Active placebos or sub-perceptual comparator designs may partially address this issue or multiple dose studies.

Third, physiological confounds, including alterations in cerebral blood flow, heart rate, and respiration can influence BOLD-fMRI signals. Incorporating concurrent physiological monitoring, e.g., through peripheral wearables or utilising complementary modalities (e.g., PET, EEG, ASL-fMRI) can enhance interpretability.

Beyond methodological issues, multimodal imaging pipelines remain costly and resource-intensive, limiting scalability, though some companies are now beginning to explore this space by utilising imaging to guide drug development and patient stratification, such as Alto Neuroscience Inc. Studies involving PET are particularly resource-intensive, with availability of particular PET ligands (e.g., [11C]Cimbi-36) also sharply limited to a small number of sites worldwide. Standardisation of methods across sites and use of open-source analysis frameworks is also a current challenge, but will be critical for reproducibility and inclusion into existing frameworks such as ENIGMA and following BEST procedures (141) will be necessary to validate such biomarkers for clinical adoption.

Together, these considerations underscore that biomarker integration into psychedelic drug development remains an evolving endeavour. Addressing these challenges will be essential for moving from correlational insight toward clinically actionable, biologically grounded theragnostic tools.

## 6 Current and future approaches

To transform psychedelic therapy into a precision treatment for addiction, biomarkers must do more than explain; they must predict, stratify, and scale. Neuroimaging markers that track changes in craving, control, and reward circuits can help identify responders, optimise dosing, and de-risk development. Large-scale data across consortium is needed to effectively validate these biomarkers utilising platforms created by organisations such as ENIGMA, the ISAM the ECNP precision psychiatry working group. Validated across multi-site and multi-centre clinical trials, these tools could support drug development, enabling the kind of targeted, biomarker-guided treatments that have revolutionised oncology, alzheimer’s and gene therapy.

Realising the vision of theragnostic psychedelic addiction medicine will require more than compelling science; it will demand collaboration across sectors. Academia must work hand-in-hand with industry, regulatory bodies, healthcare providers, funders, and patient communities to co-develop validated, scalable biomarker platforms. This includes partnerships with pharmaceutical and biotech companies developing current and next-generation psychedelics; regulatory engagement with agencies such as the MHRA, EMA, and FDA to align on biomarker qualification pathways; and integration with public and private healthcare systems to support real-world implementation.

Crucially, this vision must be properly financed. Just as oncology and neurology benefitted from multi-million-dollar precision medicine investments, building a biomarker-driven infrastructure for addiction will require dedicated funding from national research councils, philanthropic foundations, venture capital, and governmental innovation missions. The tools, data, and frameworks now exist to move beyond trial-and-error in addiction and psychiatric medicine more broadly. What’s needed next is the collective will to build it.
